# Sequencing toponymic change: A quantitative longitudinal analysis of street renaming in Sibiu, Romania

**DOI:** 10.1371/journal.pone.0251558

**Published:** 2021-05-13

**Authors:** Mihai Stelian Rusu

**Affiliations:** Department of Sociology and Social Work, Lucian Blaga University of Sibiu, Sibiu, Romania; Universidade Estadual de Maringa, BRAZIL

## Abstract

Recent scholarship in critical toponymy studies has refashioned the understanding of street names from innocent labels to nominal loci of historical memory and vectors of collective identity that are embroiled with power relations. Urban nomenclatures consist of more than mere linguistic signposts deployed onto space to facilitate navigation. Street names are also powerful signposts that indicate the political regime and its socio-cultural values. Drawing on these theoretical insights, this paper is focused on Sibiu (Romania) and explore the city’s shifting namescape in a longitudinal perspective spanning one century and a half of modern history (1875–2020). The analysis is based on a complete dataset of street names and street name changes registered across five political regimes (Habsburg Empire, Kingdom of Romania, Romanian People’s Republic, Socialist Republic of Romania, and post-socialist Romania). A series of multiple logistic regression models were carried out to determine the factors that influence toponymic change. The statistical results point out several significant predictors of street renaming: (1) the streets’ toponymic characteristics (politicized or neutral name); (2) artery rank (public squares and large avenues or ordinary streets and alleys); and (3) topographic features (a street’s size and centrality). Such a quantitative approach coupled with a longitudinal perspective contributes to the scholarly literature on place-naming practices in three major ways: firstly, by advancing an innovative methodological framework and analytical model for the study of street name changes; secondly, by delineating with statistical precision the factors that model toponymic change; and thirdly, by embedding these renaming practices observed especially after significant power shifts in the broader historical context of the changes brought in the city’s street nomenclature.

## Introduction

In the last decades, cultural geographers and scholars from other disciplinary branches of the social sciences have pumped fresh theoretical blood into the study of place-names. With the cultural turn across the social sciences and the humanities, researchers started to move away from onomastics and making etymological inroads into the origins of place-names–which dominated the traditional paradigm of place-name studies rooted in linguistics–and turned increasingly to social theory for conceptualizing the power struggles and identity stakes underpinning the politics of place-naming [1].

The new theoretical consensus expressed by the growing body of works that embrace the tenets of “critical toponymy” has compellingly shown that place-names are far from politically insignificant linguistic designators useful only to the extent that they individualize locations and indicate directions [2]. Beside these pragmatic orientational functions in facilitating navigation within a given territory, place-names are also powerful devices for the political construction of space. Imbued with symbolic meanings, place-names inscribe into the landscape political values, signify space by acting as nominal icons of identity, and constitute vectors of collective memory by which the past is brought into the present [3].

From a political perspective, place-naming and especially place-renaming are conspicuous gestures of power. Such practices constitute toponymic acts of politically appropriating the public landscape as well as nominal means of claiming authority over space [4]. Naming and renaming express authorities’ claim of symbolic ownership of the territory over which they exert their nominal jurisdiction. Moreover, (re)naming the landscape also makes an important means of legitimating power in a variety of geo-political contexts ranging from state-building [5, 6], colonialism and military occupation [7–10], decolonization [11, 12], and transition to democracy from authoritarian rule [13–15].

Renaming the landscape is often employed in the immediate aftermath of significant power shifts as a “ritual of revolution” [16] deemed to proclaim the overthrow of the former regime and to announce the triumph of a new political order. The political significance and symbolic power of place-names are rendered visible especially in the wake of regime changes, when toponymy features among the first to be targeted by the new authorities in their bid to cleanse from the landscape any reminder of the former political order [17]. In this regard, political authorities resort to renaming as an attempt to decommemorate the former regime by removing its visual symbols–materialized as ideological residua in place-names–from the public space. At the same time, renaming is used to inscribe into the landscape the ideological ethos (political symbols, heroic figures, historical memories, etc.) upholding the new structure of power.

The thorough political attention accorded to scrapping off the landscape from residual place-names is indicative of the crucial importance of toponymy in legitimizing political change. In this regard, street names in particular are powerful memorial devices of materializing the official past, and the historical narratives it is based on, onto the urban texture, and through it, into the structures of daily life [18]. From a memorial perspective, the power of commemorative street names derives from the fact that they are simultaneously toponymic “frames of memory” and “sites of remembrance” (*lieux de mémoire*) [19, 20]. Commemorative street names provide the toponymic frames for remembering the official version of the past inscribed into the urban landscape. Infused with ideological values and historical events, urban street nomenclatures are rendered into a political geography of public memory [21]. Renaming the street nomenclature thus translates into a bid to rework the collective memory upon which the legitimacy of a political regime largely rests.

Central and Eastern Europe (CEE) has been a “hotspot” of street renaming [22]. Through a series of seminal papers, Maoz Azaryahu has made (East) Berlin the analytical capital of toponymic change [13, 16, 23–25]. Other cultural geographers have documented post-socialist changes brought in urban street nomenclature in other major capital cities from the CEE region, such as Moscow [26], Budapest [27, 28], and Bucharest [17]. Other works have completed the picture by charting the toponymic transformation occurred in a variety of secondary cities such as in Łódź, Poland, St. Petersburg, Russia, Košice, Slovakia, and Timișoara, Romania [29–32]. The central thrust underpinning all these scholarly pieces was centered on assessing the nature of toponymic change brought about by the sweeping political transformations generated in the CEE region by the fall of socialist regimes and the dismantling of the Soviet Union.

The valuable contributions made by this growing body of scholarship for understanding the nature of post-socialist toponymic change across the CEE region are nevertheless affected by a series of limitations. First, the extant scholarship comprising the critical place-name studies is characterized by a dearth of quantitative approaches that employ rigorous statistical analyses. Most contributions are based on case-study approaches and provide only descriptive overviews of toponymic change inferred from a limited sample of street names (usually located in the historical centers of the cities investigated), as opposed to the entire collection of urban street nomenclature.

Secondly, scarce efforts have been made to examine toponymic transformation in the *longue durée* of modern historical development. With some notable exceptions [31], most critical place-name scholars have focused their analytical scope narrowly so as to encompass only the street renaming made following a precise regime change, usually the transition to liberal democracy in the CEE states after the demise of state-socialism during the 1989–1991 period. Such an approach falls short of situating post-socialist toponymic change in a historical perspective and thus fails to grasp the nature and scope of this recent wave of renaming in a diachronic fashion.

Studies that have taken stock of the transformative nature of urban street nomenclature over a long period of time do exist, but their geographical focus lies well outside the CEE region. Researchers have probed into the long-term changes of street names in several locations from Southern Europe (Spain, Italy) and especially from sub-Saharan Africa (Senegal, Togo, Zimbabwe). In a quantitative analysis, Carlos González Faraco and Michael Dean Murphy examined the “three wholesale transformations of street names in the Andalusian town of Almonte”, Spain. They documented how each of the three political regimes succeeding during the 20^th^ century–the Second Republic, Francisco Franco’s military dictatorship, and the post-francoist democracy–have grappled with the toponymic legacy of the former regime and imposed its own legitimizing symbols into the city’s streetscape [33]. In Italy and using advanced Geographical Information Science (GIScience) methods, Michele Tucci *et*. *al*. were able to unravel the multiple strata of street names making up the patchy and intricate “toponymic tapestry of Milan” [34].

Outside Europe, the analytical focus fell on the colonialist and postcolonialist politics of urban nomenclature. In a wide range of urban sites from sub-Saharan Africa including Dakar, Senegal, Bulawayo, Zimbabwe, Lomé, Togo, but also in Cairo, Egypt, Oran, Algeria and Kolkata, India, historical geographers have explored how the European imperial powers produced colonial places by toponymically inscribing their colonialist spatial imaginary and creating a spatial order that served their geopolitical interests. Embracing a longitudinal approach, these studies have also captured the struggle over renaming these places enacted during the postcolonial period following the 1960s, when the new independent sovereign states have engaged in processes of decolonization and nation-building [35–40]. As Brenda Yeoh has shown for the case of Singapore, toponymic cleansing coupled with the nominal inscription of nationhood were instrumental for this postcolonial political purpose [5].

This study sets out to overcome these shortcomings found in the existing scholarly literature on street name changes in the CEE region–the quantification deficit and the shortness of historical breath characterizing most approaches–by performing a statistical analysis of Sibiu’s toponymic change in a longitudinal perspective. Drawing on the analogy of urban street nomenclatures with geological strata [41], this paper aims to carry out a *toponymic stratigraphy* of Sibiu’s urban namescape. This will be accomplished in two steps: first, by unearthing the multiple layers of street names that accrued over time in the city’s urban toponymy, and second, by modelling statistically the patterns characterizing toponymic change from one historical period to the next.

Against this theoretical background, this research is driven by a series of three hypotheses that explore the role of streets’ politicized names, artery class, and topographical features in determining street name changes.

(H_1_) Street renaming is determined by the politicized nature of the streets’ toponymy. As such, streets that bear politically relevant names are more targeted for renaming than the streets with politically neutral names.

(H_2_) Street renaming varies in terms of artery class as a measure of its symbolic importance within the public road network. In this sense, boulevards and public squares are more frequently renamed than regular streets, alleys, and entrances.

(H_3_) Street renaming is also structured by an artery’s quantitative topographical features. Consequently, the probability of a street being renamed depends upon its centrality (the closer to the city center the higher probability of being renamed) and size (the larger in area the higher the odds of renaming).

Based on these considerations, this article proceeds with providing a brief overview of Sibiu’s historical background and urban development. After presenting the city selected as the geographical locus for this research, the next section points out the methodological outlines and the data collected for evaluating the empirical adequacy of the three hypotheses specified earlier. It then moves to present the results of the multiple binomial logistic regression conducted over a longitudinal dataset. The implications of these findings are explored in the concluding section of this paper.

## Historical background and urban development of Hermannstadt/Sibiu

Sibiu is one of the major cities from Romania (population ca. 150,000). The city was founded in the 12^th^ century by Germanic (Saxon) settlers brought by the Hungarian King Geza II (1130–1162) to colonize the outskirts of his empire bordering the Carpathian arc. In exchange of their military service to the Kingdom of Hungary, the Saxons who settled in Transylvania were granted significant medieval privileges. These rights consisted of economic privileges such as land use and resource exploitation, fiscal exemptions, but also political autonomy and extensive administrative jurisdiction including urban governance and legal power [42].

In 1366 the town was renamed from Hermannsdorf into Hermannstadt (after the mythical eponymous hero who founded the city, named Hermann). During the pre-modern period, as it developed into a prosperous urban center, the town surrounded itself with a complex system of fortification consisting of defensive walls punctuated by gates and stronghold towers. Inside the walls, the town was divided into four quarters (“vicinities”), each of them organized around a gate and comprising eight streets [43]. These quarters were crossed by an irregular street network characteristic of enclosed medieval European urban settlements [44]. Taxpayers’ lists kept by Hermannstadt’s municipal authorities throughout the pre-modern period point out that the towns’ streets were bearing vernacular names. These were inspired by the place’s specific topographic feature (e.g., Auf der grossen Bach/On the large brook–currently Str. Valea Mare/Large Brook Street), a nearby landmark or institution (e.g., Unter der Schulen/Under the Schools–currently Str. Alexandru Odobescu, Kirchhof/Churchyard–currently Piața Albert Huet), or otherwise made sense only to the local community in the light of its oral tradition and collective memory (e.g., Off dem Hwesruck/Dog’s Back–currently Str. Centumvirilor/Centumviri Street) [45].

An official street nomenclature exists only from 1833, when the municipality started to exert its administrative jurisdiction over the urban landscape and installed the first street name plaques on buildings’ corners [46]. In the same year, the buildings–which were first numbered in 1777 consecutively (from 1 to 1646, home to 3,420 families and a total population of around 16,000 including the 2,000 Austrian officials)–were renumbered relative to the street names [38]. These toponymic acts of naming the streets and numbering the buildings, together with other measures such as paving the streets (the town’s main commercial thoroughfare–Heltauer Gasse, currently Str. Nicolae Bălcescu–was paved entirely in 1775) were prime expressions of a process of urban modernization initiated by the municipality. During the same period, the city authorities also undertook sanitary measures against the spread of epidemics, regulated the burials, and established a police department. As historical geographers have shown for other places, together with such measures, the toponymic inscription of Hermannstadt’s landscape expressed the municipality’s modern strive to police and manage the urban space [10, 47]. This was achieved by rendering the space “legible” as a result of its rational organization. Naming the streets and numbering the buildings could be conceived of, along Foucauldian lines, as toponymic technologies of power employed by modern authorities in their bid to rationalize space and control the territory [48].

After the creation of the Dual Monarchy of Austria-Hungary following the so-called Compromise (*Ausgleich*) of 1867, Hermannstadt became part of Transleithania (Lands of the Crown of Saint Stephen) and was part of the Kingdom of Hungary [49]. This shift in the power structures within the Habsburg Empire was not without consequences on the city’s namescape. Whereas in other places in the empire, the urban nomenclatures were politicized by renaming the streetscape with honorific and commemorative names (either asserting the Austrian imperial power or celebrating the Hungarians’ national symbols), the Saxon city council of Hermannstadt chose to honor its own local history. As such, in 1872, the municipality decided to rename some of the town’s most important thoroughfares after prominent personalities from its rich tradition of political self-governance.

Nevertheless, in 1899 the town was officially renamed Nagyszeben in the context of an aggressive language policy enacted by Hungarian authorities that aimed at Magyarizing both personal and place-names [50]. The Saxon city council managed to contain the toponymic Hungarianization to the locality’s name and even resisted the pressure to add Hungarian translations to the German street names [51]. Notwithstanding its official name, Nagyszeben’s street nomenclature continued to express the city’s strong Germanic Saxon identity marked by the symbols of imperial authority. This was made further salient after the turn of the 20^th^ century, when the Austrian imperial figures were inscribed into the city’s namescape (a new street opened in 1907 was named after Joseph II, 1741–1790, Holy Roman Emperor and King of Hungary, and the city’s main avenue was renamed in 1917 after the late Franz Joseph, 1830–1916, Emperor of Austria and King of Hungary).

The intricated power struggles between the Saxon city, the Hungarian state, and the Austrian imperial authority over the urban namescape ended abruptly after the First World War. With the dissolution of the Habsburg Empire and the defeat of Hungary formalized in the Treaty of Trianon (1920), Transylvania was ceded to Romania. Nagyszeben was renamed Sibiu. In addition, the new municipal authorities ruled in 1920 that all public inscriptions–including street names–to be bilingual and subjected to a “Romanian first” linguistic policy [46]. Changes brought in the urban nomenclature through street renaming were consonant with the remaking of the ethnic demographics of Sibiu’s population. As such, in 1941 Romanians surpassed the Germanic Saxons in the city’s population structure: from a total population of 70,352, Romanians numbered 35,753 (50.82%) compared to the 28,172 Germans (40.04%) and the 4,313 Hungarians (6.13%), while the remaining 2,114 were other ethnicities (3.00%) [52].

The Transylvanian Saxons’ situation worsened dramatically during the Second World War. Romania entered the war in 1940 by joining the Axis powers and fighting alongside Hitler’s Nazi Germany. However, on August 23, 1944, a coup was staged by which Romania switched sides and joined the Soviet Union against the *Wehrmacht*. As a result, more than 70,000 of Transylvanian Saxons were arrested and sent to labor camps [53]. Shortly after the end of the Second World War, the country became the Romanian People’s Republic (December 30, 1947). The Soviet-inspired communist regime continued to persecute the German ethnics as a state policy of revenge against the U.S.S.R.’s war foes. Starting with the 1960s, throughout the Socialist Republic of Romania (1965–1989), authorities embarked on a diplomatic policy consisting of “selling” the Saxon population to West Germany. According to a secret bilateral agreement, the government of the Federal Republic of Germany accepted to pay a fee ranging from 4,000 to 10,000 Deutsche Marks for each German ethnic for whom the Romanian authorities issued an exit visa [54, 55].

These ethnic politics of exclusion that varied from brutal repression and deportation to the state business of mass emigration left a dwindled population of German ethnics in the aftermath of the democratic regime change of 1989. The first post-communist population census conducted in 1992 indicated a number of 119,462 Germans registered in Romania (0.52%) [56]. In 2002, their number dropped to 60,088 (0.28%) [57], while the latest census data from 2011 reveal a further major decrease to 36,042 (0.18%) [58]. A similar trend describes the demographic dynamics of Saxons in Sibiu: in 1992 there were 5,605 German ethnics representing 3.30 percent of the city population. By 2011, only 1,561 remained (1.06%) [58]. Despite their dwindling population, the Saxon minority, organized in The Democratic Forum of Germans in Romania (*Demokratisches Forum der Deutschen in Rumänien*–DFDR), managed to exert political control over the city and oversee much of Sibiu’s development. From 2000 onwards, the city was governed by mayors affiliated with the DFDR, including the two mandates of Klaus Iohannis, the current president of Romania. In 2007, Sibiu was designated the European Capital of Culture (together with Luxembourg), a title that consolidated the city as one of Eastern Europe’s most thriving urban centers.

## Materials and method

### Data collection and sources

This paper is based on toponymic data of Sibiu’s street nomenclature taken from five politically significant historical periods. A complete dataset of Sibiu’s street names and street name changes was constructed based on several sources. For the Austro-Hungarian Empire (1867–1918), street names were compiled from Hermannstadt’s city plans of 1875 and 1880 [59, 60]. During the short-lived Greater Romania (1918–1940), the toponymic data were taken from Sibiu’s city plan issued in 1934 [61]. For the Romanian People’s Republic (1947–1965), street names were reconstructed based on the city’s post-war plan of 1947 [62] and *Sibiu’s New Street Nomenclature* issued in 1948 [63]. The street nomenclature existing during the Socialist Republic of Romania (1965–1989) was reconstituted based on several city plans published in the early 1980s [64]. Finally, for the present, post-socialist situation, data regarding the currently existing street nomenclature were retrieved from the city council’s official records [65]. An overview of the historical sampling and the selection of the empirical materials used to construct the longitudinal dataset is provided in Table 1.

**Table 1:** Historical sampling and empirical materials.

Political regime	Historical period	Materials
**Austria-Hungary**	1867–1918	Hermannstadt’s city plan of 1875
		Hermannstadt’s city plan of 1880
**Greater Romania**	1918–1940	Sibiu’s city plan of 1934
**Romanian People’s Republic**	1947–1965	Sibiu’s city plan of 1947
		Sibiu’s street nomenclature of 1948
**Romanian Socialist Republic**	1965–1989	Sibiu’s city plans of the 1980s
**Post-socialist Romania**	After 1989	Sibiu’s street nomenclature of 2020

### Coding procedure

After obtaining the toponymic data and merging them into an integrated longitudinal dataset, the data were coded. The coding procedure involved a series of steps. The first step consisted in determining the street name changes. The street renaming was established after comparing the street nomenclatures of succeeding periods. Thus, a street name change was assessed relative to the previous historical period (e.g., a street name from the city map of 1948 was compared with the one featured on the city guide of 1934). Translations from German to Romanian that occurred in the aftermath of the First World War, when Sibiu and Transylvania become part of the Romanian Kingdom, were not recorded as street name changes.

The longitudinal nature of this study spanning a century and a half of urban development meant that we had to consider the changing nature of Sibiu’s street network. Given this dynamic of urban expansion, the new streets that were opened as the city developed were coded as such and treated as neotoponymy as opposed to renaming. These newly opened thoroughfares were taken into consideration only for the succeeding historical period when some of them could have been subjected to renaming. Similarly, the streets that disappeared from one period to another were not counted as having been renamed.

**Fig 1:**
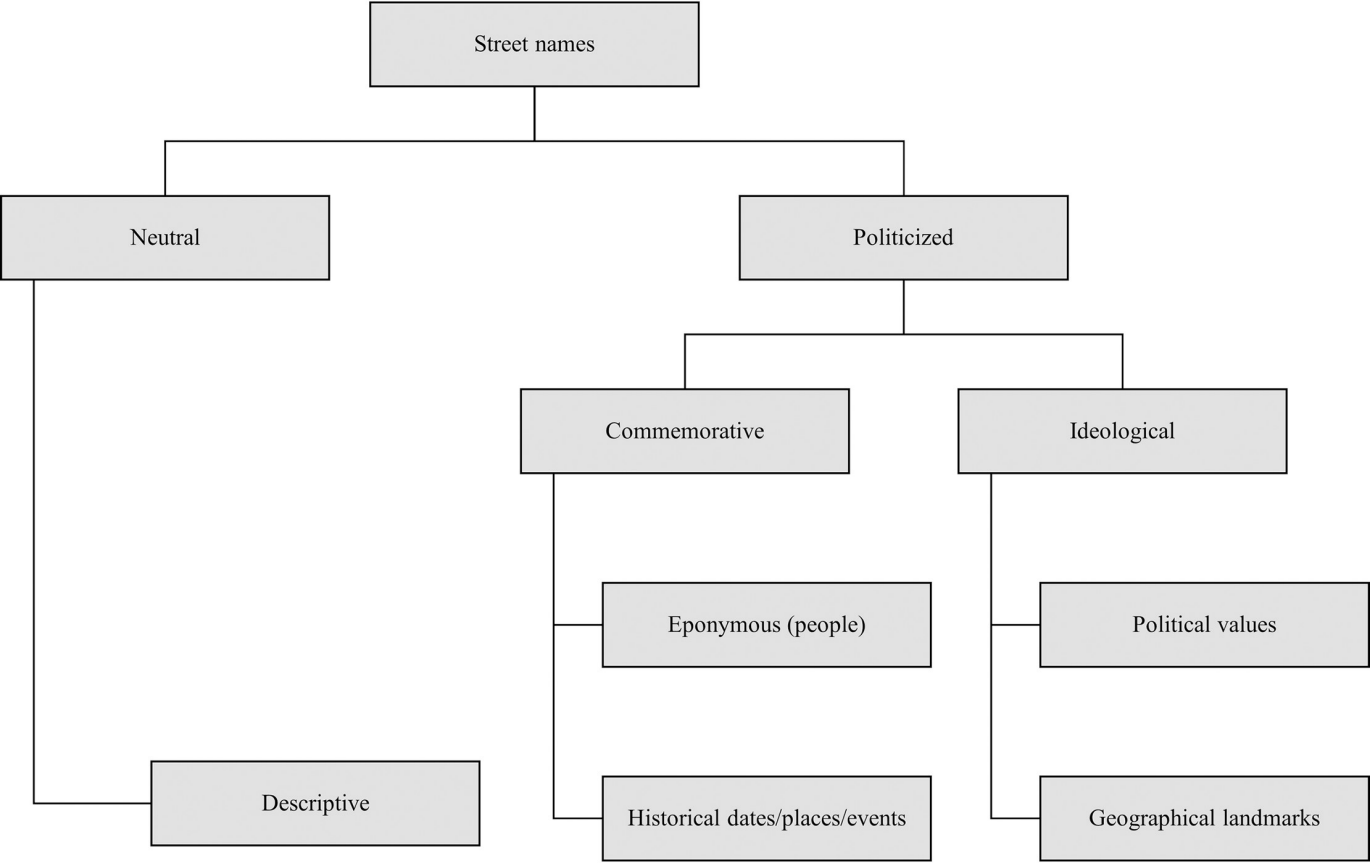
The hierarchical typology of street names (coding scheme).

Secondly, each street name was categorized in a five-fold typology as (1) “eponymous”–e.g., Str. Nicolae Iorga/Nicolae Iorga Street, after the renown Romanian historian (1871–1940) or Piața Regele Ferdinand/King Ferdinand Square, dedicated to Romania’s second monarch (1865–1927); (2) “historical date/place/event”–such as Piața 7 Noiembrie/November the 7th Square, commemorating the date of the 1917 Bolshevik Revolution; Str. Mărășești, after a battlefield from the First World War; or Str. Independenței/Independence Street, in commemoration of the Romanian War of Independence (1877–1878); (3) “political value”–Str. Egalității/Equality Street or Str. Justiției/Justice Street; (4) “geographical landmark”–Str. Moscova/Moscow Street, after U.S.S.R.’s capital, but also Str. Carpați/Carpathians Street, after the mountains that constitute a national symbol for the Romanians; and (5) “descriptive/neutral”–streets that indicate topographic features (Piața Mică/Small Square), refer to nearby locations (Str. Mitropoliei/Metropolitan Street), or bear no political relevance (Str. Florilor/Flowers Street). The classification scheme used to code the toponymic data is presented in Fig 1.

Eponymous streets (named after people) and those named after historical dates, historical places, and historical events constitute “commemorative” toponymy and are fundamental in materializing into space the collective memory–that is, the officially sanctioned version of the past–that legitimizes the contemporary political regime [18, 66]. On the other hand, the street names celebrating political values and those referring to ideologically loaded geographical landmarks could be classified as “ideological” toponymy. Taken together, these two categories–commemorative and ideological–make up the broader category of “politicized” street names, as opposed to the politically neutral ones that bear no relevant ideological meaning. Based on the coding scheme, each street was classified in only one basic category (eponymous, historical date/place/event, political value, geographical landmark, or descriptive).

Third, each road was coded according to the artery class depending on their symbolic importance within the public road network. Consequently, boulevards and public squares were classified in a first category while ordinary streets, alleys, and entrances were grouped in a second group. The categorization of an artery in one class or another was grounded on that artery’s official status relative to the epoch under consideration, as reflected in its formal name. For instance, an artery may have been designated as a boulevard in one epoch and then referred to as a street in the next. When this happened (and there were very few instances), the artery was coded as boulevard for the former period and as street for the following one.

### Method and variables

The statistical method used in this paper is multiple binomial logistic regression [67]. This is employed to model statistically the probability of the occurrence of the outcome (dependent) variable (that is, street renaming). As independent variables, the model uses three types of predictors, as follows: (1) streets’ toponymic characteristics–whether the street name is eponymous, refers to a historical date/place/event, political value, geographical landmark, or is descriptive and politically neutral; (2) artery class–whether the road is a symbolically important thoroughfare (boulevard and public square) or a less important street, alley, and entrance; and (3) streets’ topographic features–centrality and size.

Regarding the latter two characteristics, additional data were collected for determining the centrality of an artery within the network of public roads and its size. For each street, the distance in kilometers was measured from the middle of the artery to the city center consisting in the town’s main public square, that is Piața Mare/Large Square. Moreover, the size of each artery was measured by calculating the area based on official data regarding the arteries’ length and width [68].

## Results

The presentation of the findings is organized along three lines: first, the paper provides an overview of the typological make-up of Sibiu’s street nomenclature during the five succeeding political regimes covered in this analysis. Next, we move to detailing the patterns of street name changes occurred in Sibiu in a longitudinal perspective. After presenting these descriptive statistics, the analysis proceeds with discussing the results of the multiple binomial regression models. The statistical analyses were conducted using Stata 15.0 and R 4.0.0.

### Descriptive overview

#### Street name types in a longitudinal perspective

From 1875 to 2020, Sibiu’s street network expanded from 136 to 716 arteries. This steady urban growth also reshaped the toponymic structure of the city’s street nomenclature. As shown in Table 2, in the late 19^th^ century (1875), the majority of street names were descriptive (77.21%). During the 20^th^ century, as Sibiu became part of Romania, the city’s expanding network of streets was given increasingly more eponymous names. In the interwar period (1934), most streets were already named after political, historical, military, and cultural personalities drawn from the Romanians’ history. After the Second World War, the communist regime refashioned the categorial structure of Sibiu’s urban toponymy by emphasizing other types of politicized names, such as historical dates, places, and events, political values, and geographical landmarks, at the expense of descriptive names. From a structural standpoint, the city’s current street nomenclature is similar to the one existing during the second phase of Romania’s state-socialism (1980), when a fine balance was struck between politicized and descriptive names.

**Table 2:** Overview of Sibiu’s street name types by political regime.

Political regime (year of assessment) / Street name type		N	%
**Postsocialist Romania (2020)**			
	Eponymous (people)	242	33.80
	Historical dates/places/events	29	4.05
	Political values	25	3.49
	Geographical landmark	64	8.94
	Descriptive	356	49.72
	Total	716	100
**Romanian Socialist Republic (1980)**			
	Eponymous (people)	158	30.92
	Historical dates/places/events	38	7.44
	Political values	28	5.48
	Geographical landmark	34	6.65
	Descriptive	253	49.51
	Total	511	100
**Romanian People’s Republic (1948)**			
	Eponymous (people)	234	54.93
	Historical dates/places/events	26	6.10
	Political values	22	5.16
	Geographical landmark	11	2.58
	Descriptive	133	31.22
	Total	426	100
**Greater Romania (1934)**			
	Eponymous (people)	163	54.52
	Historical dates/places/events	7	2.34
	Political values	4	1.34
	Geographical landmark	4	1.34
	Descriptive	121	40.47
	Total	299	100
**Habsburg Empire (1875)**			
	Eponymous (people)	30	22.06
	Historical dates/places/events	0	0.00
	Political values	1	0.74
	Geographical landmark	0	0.00
	Descriptive	105	77.21
	Total	136	100

**Fig 2:**
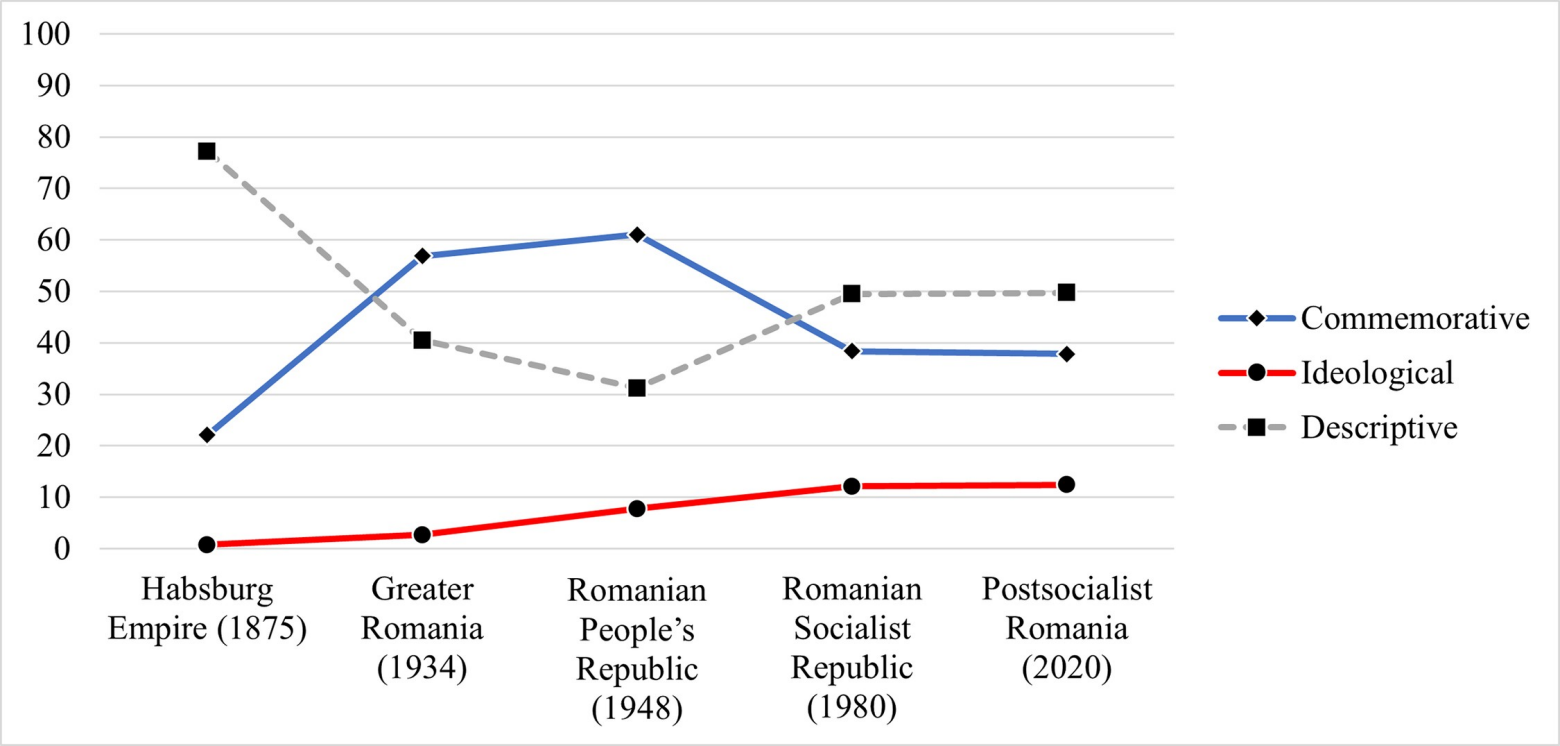
Time series of Sibiu’s street name types.

The temporal dynamics between the various types of street names becomes clear in Fig 2, which plots the time series of “commemorative”, “ideological”, and “descriptive” toponymy. It shows that the ideological names (i.e., political values and geographical landmarks), although occupying a relatively modest percentage in the overall nomenclature, have become increasingly more popular. In contrast, the commemorative names (i.e., eponymous and historical dates/places/events) have lost from their appeal, after reaching their heights during the interwar Greater Romania and the Romanian People’s Republic. What these data show is that the highest extent of toponymic politicization was reached in the regimes before and after the Second World War, which were characterized by the intense use of state-propaganda and ideological control (nationalism during Greater Romania and communism during the Romanian People’s Republic).

#### Street name changes in a longitudinal perspective

Sibiu’s street nomenclature was restructured multiple times during the period under investigation (1875–2020). The city’s streetscape was renamed successively in the aftermath of relevant political transformations and regime changes. However, as shown in Table 3, the changes brought about in the city’s urban namescape varied significantly in magnitude and scope across historical periods and political regimes.

**Table 3:** Overview of Sibiu’s street name changes by political regime.

Political regime (year of assessment)	Renamed		Unchanged		Total	
	N	%	N	%	N	%
**Post-socialist Romania (2020)**	39	7.69	468	92.31	507	100
**Romanian Socialist Republic (1980)**	103	24.29	321	75.71	424	100
**Romanian People’s Republic (1948)**	184	62.80	109	37.20	293	100
**Greater Romania (1934)**	29	21.32	107	78.68	136	100
**Habsburg Empire (1875)**	16	30.19	37	69.81	53	100

The proportion of street renaming from one historical period to another varies from a minimum of 7.69 percent after the fall of state-socialism in 1989 to a maximum of 62.80 percent registered in the post-war context after the institution of the communist Romanian People’s Republic in 1947. In between these extremes, Sibiu’s street nomenclature was partially rewritten first during the Austrian-Hungarian Empire, in the late 19^th^ century, when almost a third of the city’s street names were changed (30.19%). During the interwar period, when Transylvania and Sibiu became part of Greater Romania, a fifth of the city’s street nomenclature undergone toponymic revision (21.32%). After the post-war communist takeover of Romania brought massive changes (62.80%), another wave of toponymic transformation occurred during the 1980s, when around a quarter of Sibiu’s street names were again renamed (24.29%).

### Modelling toponymic change: Multiple binomial regression models

To understand the factors that structured the process of street name changes across these historical periods, a series of multiple binomial regression analyses were conducted for each transition from one political regime to another. The variables used to model toponymic change, alongside the descriptive statistics of the continuous predictors employed in the regression analyses (centrality and size), are provided in Table 4.

**Table 4:** Variables and descriptive statistics.

Variable	Description	Values	Measurement
**Street renaming**	Binary dependent variable referring to whether a street was renamed after each historical period	0 = Unchanged 1 = Renamed	Nominal
**Street toponymy**	Categorical variable referring to the type of name assigned to a street	1 = Eponymous 2 = Historical date/place/event 3 = Political value 4 = Geographical landmark 5 = Descriptive/neutral	Nominal
**Artery class**	Binary variable referring to the type of thoroughfare based on its importance in the public road network	1 = Boulevard, square 2 = Street, alley, entrance	Ordinal
**Centrality**	Continuous variable referring to the distance from the city center measured in kilometers	Min = 0.00 Max = 4.70 Mean = 1.94 SD = 0.98	Scale
**Size**	Continuous variable referring to the area of the thoroughfare measures in hectares (1 ha = 10,000 m^2^)	Min = 0.01 Max = 8.876 Mean = 0.410 SD = 0.725	Scale

Table 5 presents the results of the four multiple binomial logistic regression analyses. The models’ coefficient of determination varies from a Nagelkerke pseudo-R^2^ of 0.081 (Greater Romania, 1934) to a value of 0.369 (post-socialist Romania, 2020), which indicates that the regression modelling of toponymic data account roughly between 8 to 37 percent of the variation of the outcome variable (street renaming). However, statisticians have pointed out that the pseudo-R^2^ values calculated for logistic regressions are significantly lower than the coefficients of determination specific to linear regression [67]. In the light of such considerations, the logistic regression analyses built to model the toponymic change in Sibiu’s street nomenclature across historical periods seem relatively robust.

**Table 5:** Multiple binomial logistic regression models.

Dependent variable: street renaming		Greater Romania	Romanian People’s Republic	Romanian Socialist Republic	Post-socialist Romania
		(1934)	(1948)	(1980)	(2020)
		Odds ratios (z)	Odds ratios (z)	Odds ratios (z)	Odds ratios (z)
**Category**	**Predictor**				
**Street name characteristics**	Eponymous	1.011	10.150***	35.222***	8.970***
		(0.02)	(7.07)	(6.12)	(3.22)
	Historical date/place/event	1	0.617	1.115	49.016***
		(empty)	(-0.55)	(0.11)	(5.48)
	Political value	1	0.821	1.377	27.160***
		(empty)	(-0.17)	(0.26)	(4.16)
	Geographical landmark	1	2.642	1	9.204**
		(empty)	(0.94)	(empty)	(2.57)
	Descriptive/neutral (base)	1	1	1	1
		(.)	(.)	(.)	(.)
**Artery type**	Boulevard, square	1.589	1.512	8.614***	4.421*
		(0.70)	(0.64)	(3.14)	(2.14)
	Street, alley (base)	1	1	1	1
		(.)	(.)	(.)	(.)
**Topographic features**	Centrality (km)	0.435	0.514**	0.698*	0.429***
		(-1.73)	(-3.25)	(-2.36)	(-3.50)
	Size (ha)	1.845*	1.172^†^	1.662**	1.645**
		(2.13)	(0.81)	(2.70)	(2.70)
**Constant**		0.396*	1.334	0.030***	0.029***
		(-2.15)	(0.97)	(-5.67)	(-5.09)
**Observations**		132	292	409	507
**Cox and Snell R**^**2**^		0.053	0.216	0.245	0.155
**Nagelkerke R**^**2**^		0.081	0.294	0.363	0.369
**Log-likelihood**		-65.916	-157.404	-172.272	-94.901
**AIC**		141.833	330.808	358.543	205.802

Note

* *p* < 0.05

** *p* < 0.01

*** *p* < 0.001.

The results presented in Table 5 will be interpreted relative to each historical period and political regime in the following sections. Therefore, the succeeding interpretation is organized chronologically, starting with the late 19^th^ century when Sibiu was part of Austria-Hungary, moving through the interwar Greater Romania and the half of century of state-socialism, and finishing with contemporary, postsocialist Romania.

#### From Austria-Hungary to Greater Romania

Compared to the situation existing in 1875, the street name changes made by 1934 are accounted in terms of arteries’ topographic factors, as opposed to their toponymic features. In this regard, street name characteristics such as being named after persons (eponymous) did not constitute statistically significant factors that underpinned toponymic change. In contrast, it was the size that drove the process of street name change, as targeted for renaming were those streets that occupied larger areas. In this regard, a one-unit increase (1 ha or 10,000 m^2^) in the area of an artery corresponds to an increase of 84.5 percent in the odds of a street being renamed (the mean value of a street’s area is 0.410 ha or 4,101 m^2^).

It was the largest thoroughfares that were renamed after the city was incorporated in the Romanian Kingdom: Jungenwaldstrasse became Octavian Goga Avenue, after the Romanian nationalist poet and anti-Semitic politician (currently it is named Calea Dumbrăvii/Copice Road); Rotenturmstrasse was renamed B-dul Mihai Viteazul, in honor of the Romanian pre-modern ruler (currently it is named B-dul Milea, after the communist *Securitate* director who committed suicide amid the December 1989 revolutionary events); Schewisgasse was rebaptized B-dul Carmen Sylva, after the literary pseudonym used by Elisabeth of Wied, Queen of Romania (its current name is B-dul Victoriei/Victory Boulevard); the city’s main public square, Grosser Ring, was itself renamed Piața Regele Ferdinand/King Ferdinand Square, after Romania’s ruling monarch (today it was back-named to its original name, Piața Mare/Large Square).

As these examples indicate, most of the renamed streets were politicized by attributing eponymous and commemorative names to streets previously bearing descriptive names. In statistical terms, out of the 29 renamed streets, 23 (79.31%) were named after prominent personalities (eponymous), 3 (10.34%) after historical dates, places, and events, and only 3 (10.34%) received descriptive names.

#### From the Kingdom of Romania to the Romanian People’s Republic

In 1948, the size of an artery continues to count as a driving factor of toponymic change, but centrality turns out to be a more powerful predictor (Wald test z-score -3.25 for centrality in comparison to 0.81 for size, both statistically significant at alpha levels of 0.01 and 0.10 respectively). For a street that is located one kilometer farther to the city center the odds of renaming are 0.514 times the odds of surviving unchanged. Conversely, this means that a street located one kilometer closer to the city center has 1.945 times the odds of being renamed than keeping its name intact.

In addition to size and centrality, eponymous street names also become the focus of renaming. Controlling the effect of all the other predictors, the odds of renaming an eponymously named street are 10 times those of a street bearing a descriptive name. This means that a 1934 eponymous street name is 915 percent more likely to be renamed in 1948 than a descriptive street name.

Of the 184 streets that were renamed in the first year of the communist Romanian People’s Republic, 131 (71.20%) were eponymous, including the members of the royal dynasty (King Carol I, King Ferdinand I, Queen Mary, Carmen Sylva) and the pantheon of military heroes and political statesmen associated with Romania’s 19^th^ century nation-building project (Octavian Goga, the former prime-minister I. G. Duca, the First World War General Traian Moșoiu). These were replaced with Soviet leaders such as Marshal Rodion Yakovlevich Malinovsky, Andrey Vyshinsky, Veaceslav Molotov but also with socialist ideological dates and symbols (23 August [1944], 13 December [1918] in commemoration of a workers’ manifestation in Romania, Solidarity, Progress, or Moscow). Many other previously descriptive street names were politicized by renaming them after heroes, historical events, and political values associated with the new regime.

#### From the Romanian People’s Republic to the Socialist Republic of Romania

During the 1980s, eponymous names were again subject to renaming. In fact, the odds of renaming these streets named after people are about 35 times those of descriptive street names. Of the 103 streets that were renamed in this period, 96 (93.20%) were eponymous; only one was indicative of a political value, two were commemorative, and four were descriptive. With few exceptions (e.g., V. I. Lenin), Soviet leaders and heroes were purged off the urban nomenclature (I. V. Stalin, Andrei Zhdanov, Alexey Stakhanov) and replaced with mostly descriptive, politically neutral names (66 of the 103 streets, totaling 64.08 percent, were granted in 1980 descriptive names). However, in this particular context, the politically neutral names used to replace the Soviet leaders indicate that descriptive toponymy can be employed as a neutralization technique that conceals political intentions.

In addition, boulevards and squares were renamed statistically significantly more than regular streets, alleys, and entrances, whereas in the previous historical periods the artery’s status within the public road network was not relevant with regards to toponymic change. In this period, the odds of a boulevard or a public square being renamed are about 8 times those of a street or an alley, while controlling for the effects of the other predictors. For instance, Piața I. C. Frimu/I. C. Frimu Square, dedicated to a Romanian socialist militant, was renamed as Piața Aurarilor/Goldsmiths’ Square and Piața Sacco Nicolae/Nicola Sacco Square, honoring the Italian anarchist executed by the electric chair in 1927 in the United States, was renamed as Piaţa Prahovei/Prahovei Square, after a Romanian county.

#### From the Socialist Republic to post-socialist Romania

Although the street renaming made during the period of post-socialist transformation (post-1989) were the least extensive in scope (39 out of the previously existing 511 streets– 7.69%–while four of them were dissolved), they were nevertheless the most systematic toponymic transformation the city encountered in its modern history. This is because post-socialist authorities restructured Sibiu’s street nomenclature by renaming all categories of politicized street names statistically significantly more than those descriptive and politically neutral ones.

Most targeted for renaming were the street names institutionalizing into the urban landscape the historical dates, places, and events. The calculated odds of renaming this type of commemorative street names are 49 times the odds of renaming a descriptive toponymy. The political values inscribed into Sibiu’s urban nomenclature were themselves systematically scrapped (the odds of renaming the latter are 27 times those of renaming descriptive street names). Moreover, eponymous toponymy and streets named after politically loaded geographical landmarks were also changed statistically significant (the odds of renaming these categories are about 9 times those of changing the descriptive street names).

#### The topography of street name changes

**Fig 3:**
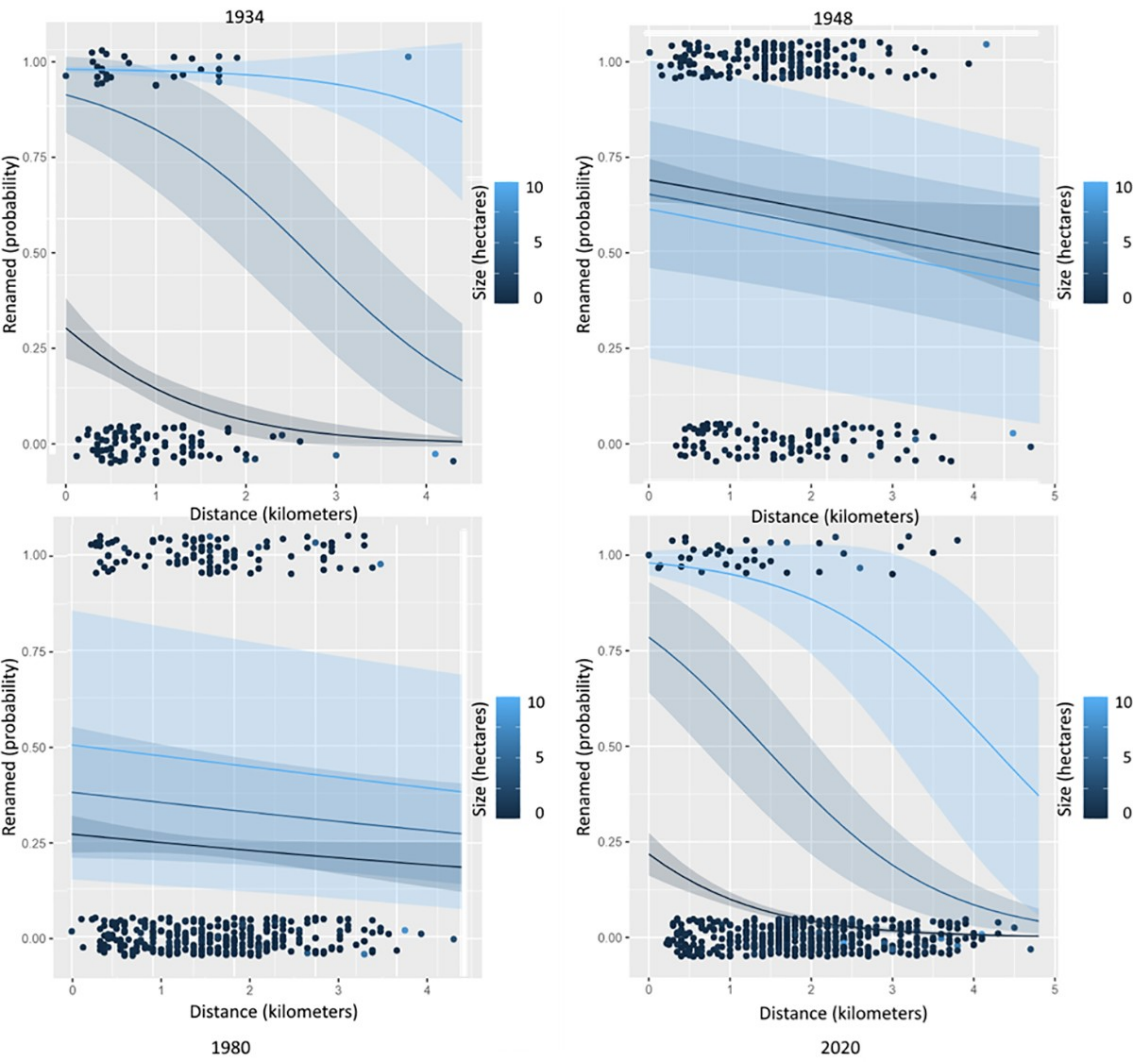
Probability of street renaming by centrality and size in 1934, 1948, 1980, and 2020.

Topographical features such as an artery’s centrality and size were statistically significant predictors of toponymic change throughout the periods included in the timespan of this analysis (1875–2020). Fig 3 provides visualizations of whether the probability of a street being renamed is influenced by its distance from the city center measured in kilometers (centrality) and area measured in hectares (size). The figures were produced using the “ggeffects” package for R [69].

The graphics were generated after modelling the outcome variable (street renaming) by distance and size, with size being held constant at 0, 5, and 10 hectares, respectively. Excluding the influence of the other predictors used in the multiple binomial regression models (Table 5), what these images show is that in 1934, 1980, and 2020 a street’s location and area impact the probability of renaming in the expected fashion (the closer a street to the center and the larger in size, the larger the probabilities of renaming). In contrast, in 1948, when the toponymic reform was of the largest scale, the size of a street had an inverse effect on the probability of renaming, in that broader thoroughfares were less probable to become targets for street name change.

**Fig 4:**
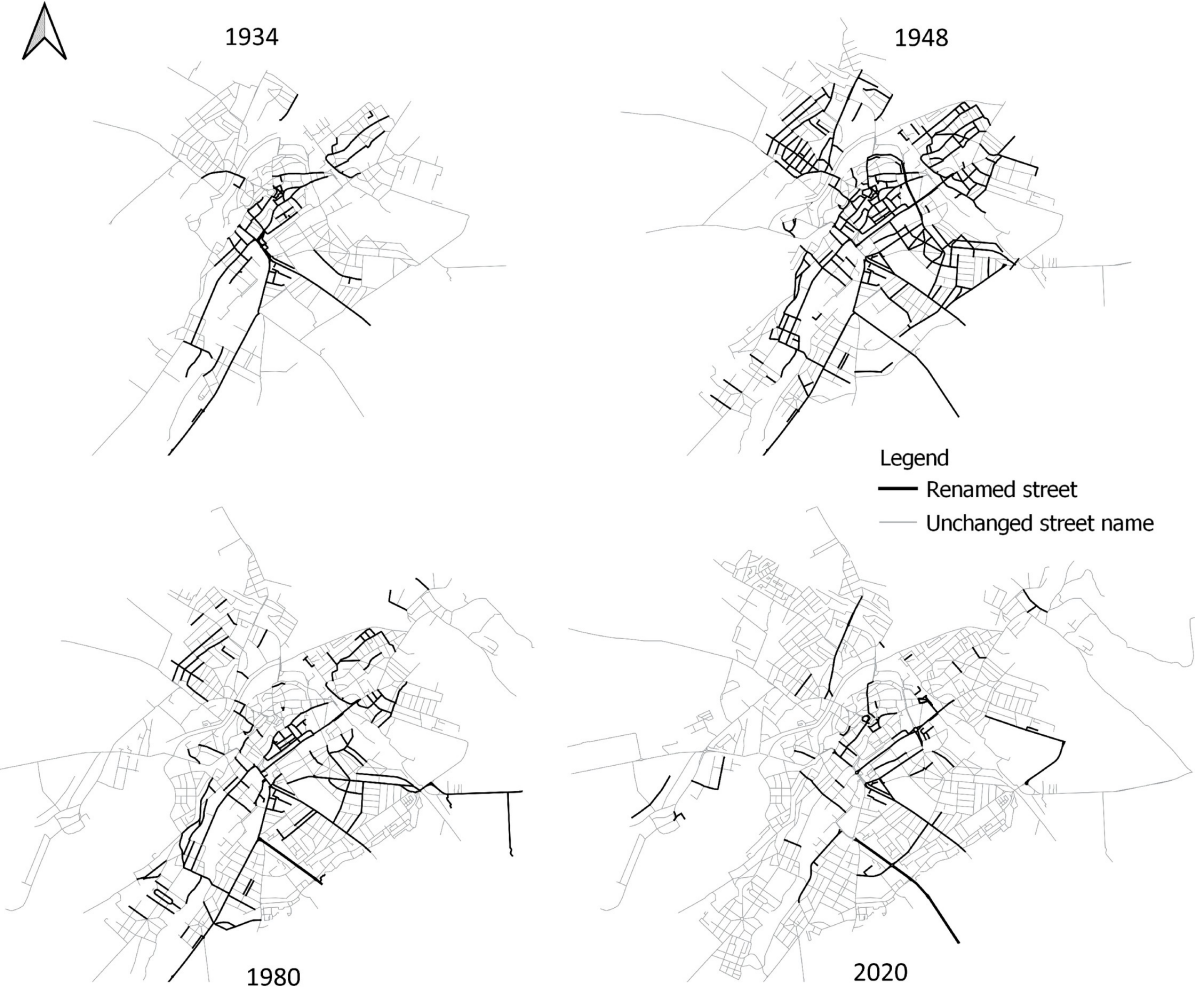
Topography of toponymic change in 1934, 1948, 1980, and 2020.

The particular geography of street name changes is rendered visible in Fig 4. This figure presents the location of the streets that were renamed starting with 1934. By locating the toponymic changes within the city plan, these images provide a visual representation of both the extent of renaming done in each of the periods covered in this study and the topographic centrality underlying toponymic change. This is the most salient in 1934, when renaming was almost exclusively concentrated in the inner core of the city. After the communist seizure of state power, the sweeping toponymic overhaul enacted by the new authorities led to the renaming of almost all the centrally located streets. However, the process also overflowed well beyond the bounds of the central area. The revisions made in Sibiu’s urban nomenclature by the 1980s and after the 1989 regime change are more loosely scattered across the city plan, but they have nevertheless affected predominantly the streets situated in the historical center.

## Discussion

Street names and urban nomenclatures are notoriously prone to change and vulnerable to toponymic revision especially in the wake of significant reconfigurations in the power structures underpinning a political regime. Sibiu’s case certainly makes no exception to this general pattern, as the results of the empirical analysis detailed earlier powerfully show. From a semiotic perspective, when examined quantitatively in a longitudinal timeframe, Sibiu’s street nomenclature reveals itself as a *structurally unstable signifier*. This peculiar quality of urban namescapes, that was documented in a variety of places around the world, is a direct outcome of the toponymic politicization of space through the assignment of ideologically meaningful names to streets previously bearing descriptive and non-political names.

The Dual Compromise of 1867 opened the flood gates for the politicization of street nomenclature through renaming the urban toponymy throughout the Austro-Hungarian Empire. Sibiu was among the first Transylvanian cities to rework its namescape along honorific and commemorative lines. In Sibiu, this process of politicizing the urban toponymy started in 1872, when the first eponymous names celebrating the city’s political elite were inscribed in the street nomenclature. Other places from Transylvania followed suit, with city councils bringing large-scale changes through renaming in Arad– 1880, Brassó/Brașov and Marosvásárhely/Târgu Mureș– 1887, Kolozsvár/Cluj– 1899, and Temeswar/Timișoara– 1902 [70].

During the 19^th^ century, European cities rendered their descriptive and vernacular street names in official urban toponymies. In contrast to other cities from the Habsburg Empire, which during this period transformed their street nomenclature by renaming the descriptive toponymy with honorific and commemorative names, the leadership of Saxon towns from Transylvania consistently chose to make official their vernacular toponymy and to resist politicizing their urban namescapes. Paradigmatic in this regard is Brassó (Krondstadt/Brașov), whose Saxon city council made only two changes to the street nomenclature as late as 1887. By renaming two important thoroughfares after the 16^th^ century mayoress Apollonia Hirscher and the 17^th^ century mayor Michael Weiss, the municipality chose to affirm its own local history rather than to celebrate imperial personalities. Hermannstadt was the only one to defy this Saxonian norm: in 1872, the city renamed eleven central streets after local notables while two years later, in 1874, another eight streets were renamed in the suburbs [18, 70].

The changes made in the Sibiu’s namescape after Transylvania became part of Romania in the aftermath of the First World War must be situated in a broader process of Romanianization [71]. As part of the ethnic struggle to assert the Romanian national element in the newly incorporated territories, Romanian authorities have issued normative linguistic policies. In this regard, a governmental disposition ordered local councils “to give Romanian names to the streets: historical names, referring to the royal family and the country’s great men, of those who lived in the past as well as of those who have made Greater Romania” [72]. In the interwar period, the Romanianization process turned increasingly radical not only with regards to the renaming of streets. In 1936, the policy of bi- and trilingual public signage applied to street plates among other inscriptions displayed in the public space was abandoned. From now on, all public signage had to bear exclusively Romanian names and the use of the former, German and Hungarian names, was forbidden [72].

Relevant comparisons could be made in terms of the extent of street name changes occurred after 1918 between Sibiu and other Transylvanian cities for which such toponymic data are available. In Alba Iulia (formerly Gyulafehérvár), of the 33 streets documented in the city plan of 1910, only five survived with their name unchanged in 1928 [73]. The extreme scope of toponymic change (84.85%) can be explained given Alba Iulia’s special place within Greater Romania’s symbolic geography, as “the City of the Great Union” (*Orașul Marii Uniri*). It was here that the Resolution of the National Assembly was signed on December 1, 1918, proclaiming Transylvania’s unification with Romania.

In the Saxon town of Sebeș (formerly Mühlbach), the extent of renaming reached 76.19 percent (32 out of the 42 pre-existing streets) [74], while in Târgu Mureș (formerly Marosvásárhely), where the Hungarians constituted in 1910 up to 90 percent of the local population, the scope of toponymic change was less extreme (91 of the 151 pre-existing streets– 64.90%) [72]. Compared to these cases, Sibiu’s situation is indicative of a much milder process of toponymic Romanization (29 streets were renamed by 1934 from a total of 136 pre-existing streets– 21.32%).

Sibiu’s urban namescape underwent a genuine toponymic revolution after the establishment of the communist Romanian People’s Republic. City authorities rushed to rename the streetscape as early as 1947, and then again in 1948. Overall, 184 of Sibiu’s 293 streets were renamed (62.80%). This situation contrasts markedly with the rather modest scale of the toponymic reform implemented in Romania’s capital city. Based on the broad theoretical consensus that “capital cities are special places” that occupy central positions in a country’s symbolic geography [75], Bucharest’s street nomenclature should have been the prime target of revision. In general, “the space of a capital city serves as a stage for the ‘commemorative vigilance’ of a nation, that is, for the physical and symbolic maintenance of its memory” [76]. Following political unrest and regime change, it also becomes the subject of extensive recasting. Empirical data present, however, a completely different picture. In Bucharest, between 1946 –when a major avenue was renamed as Boulevard Generalissimus Stalin–and the end of 1948, only 117 of the capital city’s 3,000 streets were renamed (3.90%). Duncan Light stated that “in the first year following the proclamation of the People’s Republic over 150 streets in Bucharest were renamed” [17]. A closer look reveals that during 1948, only 85 streets were renamed. Another set of 80 streets were either attributed names since they were not bearing any or were provisionally named with Latin letters and renamed with proper names.

The percentage did increase during the following years, when the new city leadership started to rename the urban landscape after the pantheon of heroes drawn from the Romanian socialist movements’ history of class struggle (e.g. Ilie Pintilie, I. C. Frimu, etc.) [17]. Such a discrepancy between the center and the periphery observed in the capital city and Sibiu respectively with regards to toponymic change after the communist seizure of state power substantiates the critiques of the taken for granted presupposition according to which the capital city is representative of the territory [77]. The results obtained in this paper further challenges the capital-centrism encroached into the study of place-names and calls for more attention being paid to regional, secondary cities as well as to the wider territories beyond the capital city.

Throughout the Socialist bloc in the CEE region, Stalin’s death in 1953 and the de-Stalinization process triggered after Nikita Khrushchev’s 1956 denunciation of the personality cult, entailed a broad reconsideration of the Soviet past. In Romania, the shock waves of the 20th Congress of the Communist Party of the Soviet Union were felt when Stalin’s statues disappeared from public squares (in Bucharest, Stalin’s monumental statue was toppled in 1962 and the place where it was mounted, previously dedicated to the Generalissimus Stalin was renamed as Piața Aviatorilor/Aviators’ Square; in the same year the statue of Stalin erected in Brașov was also discretely removed). In 1960, Brașov reclaimed its pre-communist identity, after a decade of being officially named the City of Stalin (*Orașul Stalin*) [78].

The re-appropriation of the national ideology started during the mid-1960s further accelerated Romania’s alienation from the Soviet Union. In Sibiu, Stalin Street/Strada Stalin was renamed after Nicolae Bălcescu, a prominent 1848 Romanian revolutionary, in as late as 1970. Overall, the extent of renaming made in Sibiu during this second phase of state-socialism (24.29%– 103 streets renamed from a road network numbering 424 streets) is similar to the case of Brașov, where by 1966 the local authorities have changed the names of 126 of the 439 pre-existing streets (28.70%).

The regime change of 1989 and the post-socialist transformations that followed suit set in motion a process of de-commemoration. However, the urban landscapes of Romania’s post-socialist cities were not thoroughly purged from residual names inherited from the former regime. The relatively small scale of toponymic revision made after 1989 has led scholars to shift their attention from street name changes toward “toponymic continuity” and to examine “the ongoing lives of street names” [79, 80].

Sibiu’s post-socialist toponymic change is quantifiable at 7.69 percent (39 from a total of 507 streets were renamed). The results obtained for Sibiu are comparable with other Romanian cities. In Transylvanian cities, the scope of post-socialist toponymic change ranges from 8.20 percent in Brașov to 12.40 percent in Cluj-Napoca to a record-high 25.99 percent in Timișoara [81]. In Bucharest, changes brought to the urban namescape after 1989 were even smaller, with only 288 streets renamed of the capital city’s 4,369 streets (6.59%) [17].

## Conclusions

This paper examined the patterns of street name changes in Sibiu, Romania, along a longitudinal perspective covering the last century of a half of modern history (1875–2020). Moving past the analytical default established in the critical toponymies scholarship–which consists in assessing street name changes after a single regime change through descriptive statistics–this paper provided a historically comprehensive account of toponymic revision grounded on a series of logistic regression models. Such a longitudinal approach based on the complete dataset of street names and street renaming, coupled with the use of multivariate statistical techniques (multiple binomial logistic regression) allowed us to make visible the determinants of toponymic change across each of the historical period that was investigated.

The statistical modelling of these comprehensive data collected for this study revealed three main categories of factors that structured the process of street renaming in Sibiu starting with the second half of the 19^th^ century onwards. A first category consists of characteristics pertaining to the street names. In general, politicized street names (that is, honorific and commemorative nomenclature dedicated to people, historical dates, events, or places, but also urban toponymy glorifying political values or indicative ideologically-loaded geographical landmarks) are more likely of being renamed that descriptive and politically neutral street names.

Secondly, the artery class to which a thoroughfare belongs is another predictor of toponymic change. Urban road networks are stratified into various types of categories, ranging from alleys, entrances, and cul-de-sacs, through regular streets, to boulevards and public squares. Confirming the theoretical expectations, the statistical analyses carried out in this paper have shown that boulevards and squares are more prone to become targets of renaming in comparison to the regular streets and alleys.

Lastly, topographic features also play an important part in the process of toponymic change. In general, centrality and size matter, so that streets that are located closer to the city center and occupy larger areas are more likely to be renamed following a significant power shift in the political regime. It is only when the scope of toponymic upheaval reaches exceptional proportions (such as was the case in the Romanian People’s Republic in 1948) that these factors become largely irrelevant.

Overall, the three hypotheses tested in this paper are generally well supported by the empirical findings. A detailed account of their empirical assessment is provided in Table 6.

**Table 6:** Overview of the empirical assessment of research hypotheses.

No.	Hypothesis	Empirical assessment
**H**_**1**_	Street renaming is underpinned by the politicized nature of the streets’ toponymy.	Partially supported by data for 1948 and 1980. Strongly supported for the post-socialist period (2020)
**H**_**2**_	Street renaming varies in terms of artery class as a measure of its symbolic importance within the public road network.	Supported for 1980 and 2020, rejected for 1934.
**H**_**3**_	Street renaming is structured by an artery’s topographical features	Both centrality and size matter across historical periods at different levels of statistical significance.

The main limitation of this work consists in the case-study approach focused on examining the patterns of street name changes in a single place. Such a methodological strategy restricts the comparability of the findings. Further work is needed for addressing the methodological issue that limits the comparability of this paper. A direction for future research would be to expand the approach to a multiple case-study design. Such an approach promises to provide better insights into the historical dynamic of street name changes across a broader region that would enhance our understanding of the politics of urban namescapes.

## Supporting information

S1 Dataset Sibiu street name changes (1934, 1948, 1980, 2020). (SAV)
